# Quantification of gastric emptying with magnetic resonance imaging in healthy volunteers: A systematic review

**DOI:** 10.1111/nmo.14371

**Published:** 2022-03-27

**Authors:** Davide Bertoli, Emily Steinkohl, Esben Bolvig Mark, Christina Brock, Asbjørn Mohr Drewes, Jens Brøndum Frøkjær

**Affiliations:** ^1^ Mech‐Sense Department of Gastroenterology and Hepatology Aalborg University Hospital Aalborg Denmark; ^2^ Department of Clinical Medicine Aalborg University Aalborg Denmark; ^3^ Mech‐Sense Department of Radiology Aalborg University Hospital Aalborg Denmark

**Keywords:** gastric emptying, magnetic resonance imaging, quality improvement, stomach, systematic review

## Abstract

**Background:**

Several magnetic resonance imaging (MRI) protocols have been used to assess gastric emptying (GE) with MRI. This systematic review summarizes the current literature on the topic. The aim was to provide an overview of the available imaging protocols and underline the items that appear most agreed upon and those that deserve further investigation.

**Methods:**

According to PRISMA guidelines, two independent reviewers conducted a systematic literature search with a pre‐specified strategy in different databases. Peer‐reviewed articles that utilized MRI techniques to assess GE in healthy volunteers (HVs) were included. The quality and the outcomes of the studies were reported and analyzed.

**Key Results:**

The literature search yielded 30 studies (531 HVs, weighted mean age 27.4, weighted mean body mass index 23.0 kg/m^2^), T2‐weighted sequences, balanced turbo field echo, and balanced gradient echo were evenly utilized, with volunteers in the supine position (74% of the studies). After overnight fasting, both liquid (56%) and mixed (44%) meals were equally utilized. Segmentation of the volumes was predominantly performed manually (63%) with a reported mean T50 ranging from 7 to 330 min.

**Conclusions & Inferences:**

As observed in this systematic review, MRI is a flexible tool for assessing GE. Different protocols were analyzed, showing an equal capacity to assess the GE. However, many items in these protocols still require further investigation to obtain a common standard and increase this assessment quality.

## Graphical abstract

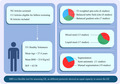

**Key Points**

- Several MRI protocols have been developed for assess the gastric emptying process in the last decade. This paper aims to provide an overview of the topic and underline the items that deserve further investigation.
- This systematic review showed that different protocols were equally capable of assessing the GE but that a common standard has not yet been defined.
- Further studies in this field are still necessary to define high‐quality clinical guidelines for this assessment.

## 1 INTRODUCTION

In the last decade, a growing interest in gastric volume and function assessment has been observed in the current literature arising from many different fields.^1^, ^2^, ^3^ In particular, the measurement and visualization of the gastric emptying (GE) process received attention in clinical and pharmacological studies due to the rising incidence of diabetes and obesity.^4^

While the commonly accepted gold standard for the measurement of GE is scintigraphy,^5^ its role has been disputed due to its shortcomings as, for example, complexity and radiation exposure.^6^ Different research groups have estimated the GE process using magnetic resonance imaging (MRI). MRI was chosen not only due to its non‐invasive nature, where ionizing radiations are avoided, but also due to its ability to provide morphological information about the stomach, to assess motility and accommodation, and to distinguish between the different components of gastric content, that is, an ingested meal, gas, and gastric secretions. Usually, an MRI study of the GE comprehends a fasting period, the consumption of a meal, the recording of multiple image series, the partitioning of voxels into meaningful sub‐volumes (segmentation), and finally, the calculation of GE parameters. Thanks to a decade‐long effort and technological developments in this field, different MRI protocols for estimating GE have been developed.

The gap between MRI and scintigraphy was vast in the past, especially in the clinical field, but with the introduction of semi‐ and fully automatic segmentation methods, this is getting progressively narrower. These segmentation methods counterbalance one of the most significant drawbacks of MRI: the need for manual, time‐demanding gastric volume analysis, resulting in more expensive examinations vulnerable to subjective evaluations.^7^ Another major and probably underestimated drawback of MRI studies for the assessment of GE is the lack of a definite standardized protocol, resulting in centers using dissimilar meal types, MRI sequences, image segmentation, GE calculation methods, and reporting GE with different quantitative outcome measures. While this could be partially explained by their almost experimental nature, it must be pointed that, to date, it has never been attempted to reach a possible consensus. At last, it is essential to underline that, while being accepted as the golden standard, scintigraphy faces some of the same drawbacks as MRI, with normal values established for different meals and with many centers using unvalidated ones.^5^

The purpose of this systematic review is to provide clinicians and radiologists with a clear overview of the existing literature on the subject. We analyzed and interpreted different MRI studies of the assessment of GE in adult human healthy volunteers (HVs), underlining the items that appear the most agreed upon and those which would deserve further investigations. We anticipate that this review will be especially useful to clinicians interested in adopting MRI in clinical practice for evaluating GE, as this examination will address many critical factors and variables. Finally, we suggest a few points to ponder to those already working with this technique, including clinical research, underlining the impact of the lack of consensus in this field.

## 2 METHODS

This systematic review followed the Preferred Reporting Items for Systematic Reviews and Meta‐Analyses (PRISMA) guidelines.^8^ The protocol has been registered on PROSPERO prior to the literature search (Reg. number: CRD42020192377).^9^

Eligibility criteria are listed in Table 1. We only included original articles published within the last 10 years to limit the technological heterogeneity between the included studies. In addition, only papers enrolling HVs, as the main participant group or as a comparative control group, were included in the analysis to decrease the possible confounding effects of pathological conditions on the outcome parameter.

**TABLE 1:** Criteria for inclusion of articles in the systematic review

Inclusion criteria
Article characteristics
Published within the last 10 years
English language
Has to report original data: original articles are included, whereas reviews, letters, or other similar sources are excluded
Population or sub‐population characteristics
Human
Adult (>18 years)
Has to contain at least a healthy subgroup
Reported outcomes
Gastric emptying should be reported as half‐emptying time (T50), or other meaningful measures (Volumes over time, emptied percentage over time)
Outcomes should be reported numerically and not exclusively in graphical form

To provide the highest strength to our search strategy, a comprehensive search of Pubmed, Web of Science, Embase, and Google Scholar (first 200 results) was conducted as suggested by a recent study.^10^ Additional data were retrieved from Cochrane Library and OpenGrey. Original articles published between January 2010 and June 2020 were retrieved from these databases (last search June 11, 2020), with the assistance of a university librarian.

The terms “Magnetic Resonance Imaging” and “Gastric emptying” and their synonyms were used. The entire electronic search strategy for the databases mentioned above can be found in Appendix 1. The search process results were imported in Endnote (Clarivate) to remove duplicates.

### 2.1 Study inclusion and data extraction

Two authors (D.B. and E.S.) assessed all publications independently with a third experienced radiologist (J.B.F.) to resolve conflicts if no agreement could be reached by consensus. The web app Rayyan (Qatar Computing Research Institute [Data Analytics]) was used for the inclusion process.^11^

A two‐level inclusion process was used: Firstly, with a title and abstract review (level 1), and secondly, with a full‐text review (level 2). All studies that could not be excluded at level 1 were retrieved in full text. When the inclusion criteria were ambiguous, the article was included for further analysis at level 2. The main requirement for inclusion at level 2 was the description of the MRI technique utilized to assess GE in HVs. When population overlap was suspected between studies of the same author, the most unequivocal report was chosen. No authors were contacted for further inquiry.

If the paper was included in the study, data were independently extracted, collected, and managed by two authors (D.B. and E.S.) using standardized piloted forms in REDCap (Research Electronic Data Capture); see an example in Appendix 2. The extracted data comprehended: (i) general study information including author, publishing journal, country of origin, and sources of funding; (ii) study characteristics including study design, inclusion and exclusion criteria, HVs selection method; (iii) participant characteristics including demographic data; (iv) intervention and setting including fasting protocol, MRI protocol, meal characteristics; (v) outcome characteristics including primary and secondary outcomes; (vi) characteristics for quality and applicability evaluations.

### 2.2 Risk of bias of individual studies

The QUADAS‐2 tool (Quality Assessment of Diagnostic Accuracy Studies) was utilized to assess study quality and relative risk of bias.^12^ The instrument was tailored to develop a review‐specific tool that showed fair agreement between authors: more relative weight was given to the domains 1, 2, and 4 (patient selection, index test, and flow and timing) as they were identified as the most susceptible to bias and applicability concerns. Domain 3 (reference standard) was not utilized for the quality assessment in our systematic review. While the scintigraphic assessment of GE would appear to be the optimal reference method, only a few studies included a direct comparison, and therefore, this domain was not included in the risk analysis.

### 2.3 Risk of bias across studies

For assessing the risk of bias across studies, the guidelines of the Cochrane Handbook for Systematic Reviews of Intervention were followed.^13^ Potential financial and non‐financial conflicts of interest, together with different types of reporting bias, were evaluated. Results of this assessment were reported mainly narratively due to the number and nature of the included studies.

### 2.4 Performance measure and synthesis of results

The stomach emptying is a complex process characterized by highly coordinated responses to the presence of a meal in the stomach. Studies have reported this process by the use of different outcome parameters. Some of these can only be obtained through direct observation, for example, emptied percentage over time, series of stomach volumes at different fixed time points (usually reported in ml), or as the area under a stomach‐volume/time curve (AUC, calculated in ml * min). Others, like the stomach's half‐emptying time (T50, calculated in minutes), which refers to the time taken for the stomach volume to reach 50% of its initial post‐meal value, can also be obtained through the use of mathematical models. While a specific overview of these models is outside the scope of this review, it is worth mentioning that the T50 is heavily dependent on the model utilized to retrieve it. Different models have been proposed in the literature, ranging from the first established method introduced by Elashoff et al.,^14^ to more complex models of data fitting as power exponential models (PowExp), linear exponential models (LinExp),^15^ or two‐component nonlinear mixed effect models.^16^ These mathematical models are defined not only by their governing equations but also by their assumptions and constraints, which may result in an oversimplification of the emptying process and loss of physiological information. Furthermore, the model's performance is also dependent on the amount of data fed to it and on the complexity of the model, which could result in under‐ or overfitting.

In this systematic review, T50 was adopted as the preferred outcome parameter because it is easily obtained from different reports and is intuitively easy to understand, interpret and compare. Consequently, studies reporting AUC instead of T50 or stomach volumes’ measurements were excluded (four studies).

A formal meta‐analysis was not performed due to the heterogeneity and relatively small number of the included studies. We did, however, report the MRI GE outcomes in comparison with scintigraphic normal values, as described in the literature.

### 2.5 Handling of missing data

#### 2.5.1 Missing characteristics of meals

When the information on meals’ characteristics was missing, the product information sheet was retrieved from the producer if the product was still available on the market.

In articles reporting only the fat percentage or fat calories, its weight in grams was calculated with 1 g Fat = 9 kcal. To convert water volume to weight, we assumed all the studies were performed under standard conditions where 1 L of water = 1 kg. The items generated with these conversions are underlined in Appendix 4.

#### 2.5.2 Calculation of T50

In articles only reporting GE through volume datasets, the T50 was imputed using the free online tool ‘apps.menne‐biomed.de/gastempt/’, utilized in other relevant studies in this field.^15^ The application permits, given a series of gastric volumes over time, to approximate an emptying curve and estimate the T50. The T50 calculated with this application are underlined in Appendix 4.

### 2.6 Comparison with scintigraphy

As previous studies suggested that MRI and scintigraphy could accurately assess gastric volumes,^17^ two consensus articles were chosen to define the normal scintigraphic values, which will be used as “standard” reference. A combination of the terms “Gastric emptying scintigraphy” and “Gastric emptying” and their synonyms was used to retrieve these articles. As the first search did not include articles where the emptying process was measured in response to a liquid high‐calorie meal, another article was obtained and included through the combination of the terms “Gastric emptying scintigraphy,” “Gastric emptying,” and “High calorie” or “Fat” was used in the search.^18^

### 2.7 Statistical analysis

The degree of agreement in the inclusion process was determined with Cohen's kappa coefficient (*κ)*.^19^ The kappa results will be reported following the definition of Landis & Koch, see Table 2. Statistical analyses were performed using STATA version 16 (StataCorp LP).

**TABLE 2:** Interpretation of Cohen's κ agreement scores

Cohen's κ value	Interpretation of the agreement score
0	No agreement
0.1–0.20	Slight agreement
0.21–0.40	Fair agreement
0.41–0.80	Substantial agreement
0.81–0.99	Near‐perfect agreement
1	Perfect agreement

## 3 RESULTS

### 3.1 Literature search

**FIGURE 1:**
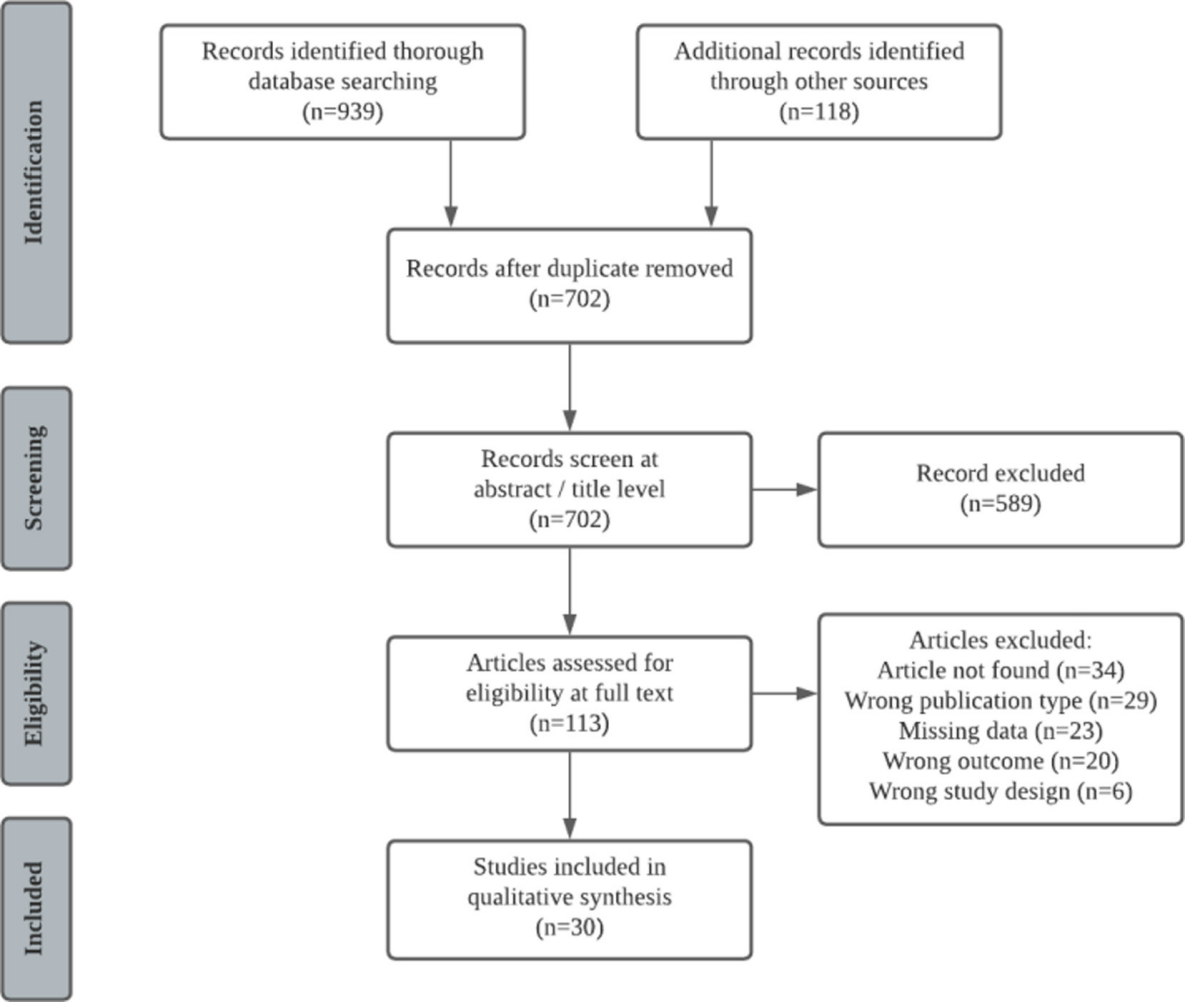
PRISMA (Preferred Reporting Items for Systematic Reviews and Meta‐Analyses) flowchart of the inclusion process

The literature search in Pubmed, Embase, Web of Science, and Google Scholar yielded 939 results; an additional 118 were retrieved from Cochrane Library and OpenGrey. After duplicate removal, 702 articles were included to be screened for eligibility. Of them, 113 articles were eligible for full‐text screening, and 30 articles were included for detailed review.^1^, ^2^, ^3^, ^4^, ^15^, ^20^, ^21^, ^22^, ^23^, ^24^, ^25^, ^26^, ^27^, ^28^, ^29^, ^30^, ^31^, ^32^, ^33^, ^34^, ^35^, ^36^, ^37^, ^38^, ^39^, ^40^, ^41^, ^42^, ^43^, ^44^ The agreement between the authors in the inclusion process, depending on the screening phase, ranged between 94.6% (Cohen's *κ* = 0.73) at level 1 and 80.5% (Cohen's *κ* = 0.61) at level 2, showing substantial agreement in both. The inclusion process is illustrated in Figure 1.

### 3.2 Risk of bias and applicability concerns

A fair agreement between the authors (Cohen's *κ* = 0.27) was observed at this stage.

**FIGURE 2:**
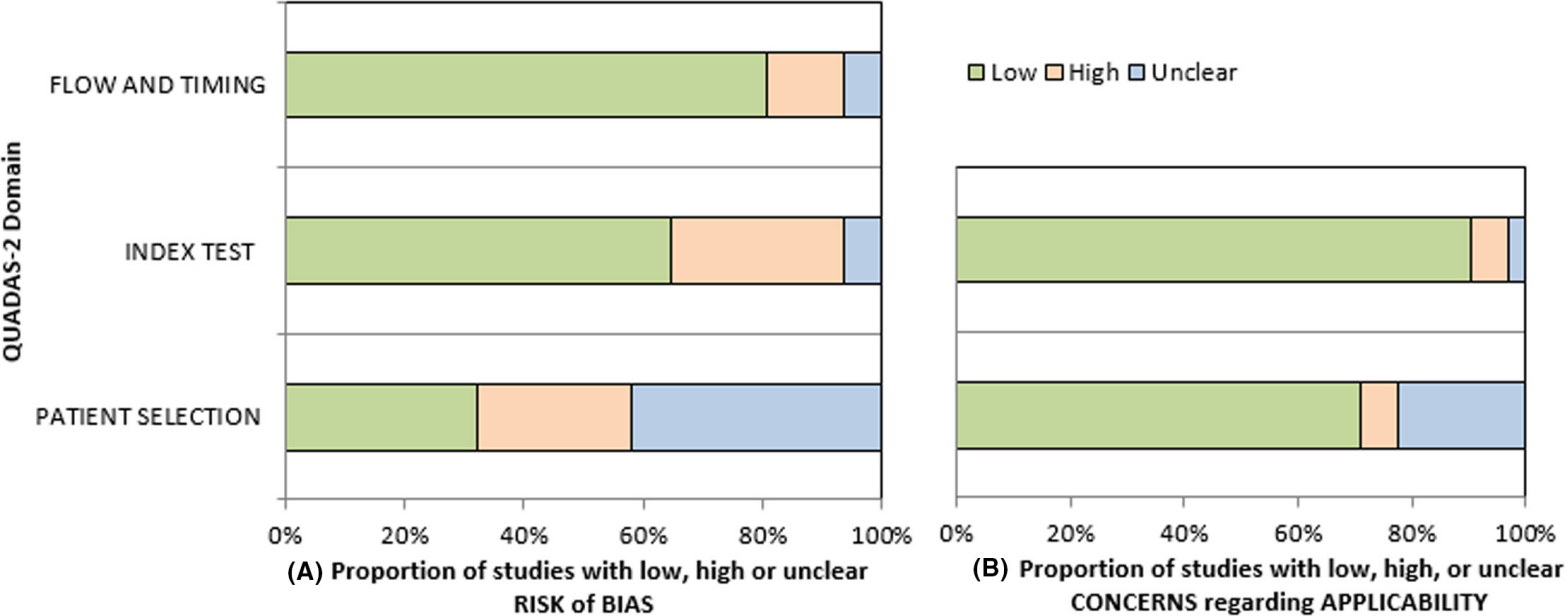
QUADAS‐2 (Quality Assessment of Diagnostic Accuracy Studies) quality assessment tool. Each domain (patient selection, index test, flow, and timing) is graded with a Low/High/Unclear risk of bias (A) and with a Low/High/Unclear applicability concern (B)

The QUADAS‐2 tool showed a low risk of bias ranging from 80.6% (flow and timing domain) to 32.2% (patient selection domain); see Figure 2A. Applicability concerns analysis showed an overall low level of concern, from 70.9% (patient selection domain) to 90.3% (index test domain); see Figure 2B. The evaluation of every single item for the included studies can be found in Appendix 3.

### 3.3 Risk of bias across studies

Financial disclosures were reported in most of the included studies (24 studies, 80%). Twenty (83%) were founded by public national or international grants. No financial conflicts of interest were reported. The risk of publication bias is difficult to assess, as the main outcome measure of our interest, the T50, was not necessarily the primary outcome measure of the included studies. In the light of this observation, selective (non‐)reporting biases were also judged non‐influential in our systematic review.

### 3.4 HV demographic and selection

The analyzed 30 studies included a total of 531 HVs (mean 18 subjects in each study, range 5–73), weighted mean age 27.4 years (range 18–65 years), weighted mean body mass index (BMI) 23.0 (range 20.8–26.4 kg/m^2^), gender ratio: male versus female 62%/38%. The vast majority (28 studies, 93%) of the included studies enrolled between 5 and 34 HVs, while two enrolled 50 and 73 HVs, respectively.^25^, ^26^ These two studies alone included 23% (123 of 531 HVs) of the subjects of our analyses. It was only specified in 12 of the 30 studies (40%) that a consecutive or random sample of HVs was enrolled. In most of the included studies, potentially disturbing factors to the gastrointestinal functions (acute or chronic gastrointestinal diseases, surgical procedures of the gastrointestinal tract, medication potentially interfering with gastrointestinal functions) were evaluated either in the inclusion or exclusion process (18 studies, 60%). Restrictions based on the cardiorespiratory or neurological status were enforced only by a few studies (five studies, 17%).^1^, ^3^, ^33^, ^39^, ^45^ In only one study, the health status of the HVs was assessed by physical evaluation^36^; otherwise, it was assessed by self‐reporting. Half of the studies had age or BMI restrictions (15 studies, 50%).

### 3.5 Study protocols

A fasting protocol was reported in 27 (90%) of the included studies. Most integrated an overnight fasting period, with alcohol restriction and caffeinated drinks 24 h before the baseline scan. The fasting restrictions ranged from 3 h (or 1 h for water intake)^31^ to 12 h (8.00 p.m. to 8.00 a.m.).^34^, ^37^, ^38^, ^43^ Subjects were also instructed to avoid strenuous exercise in 13 studies (43%).^3^, ^24^, ^26^, ^27^, ^30^, ^32^, ^34^, ^35^, ^37^, ^41^, ^42^, ^43^, ^46^

Most MRI protocols included a baseline scan, intake of a meal, and two or more scans (up to 21) to evaluate the GE. The length of the GE studies ranged between 30 min^29^, ^33^ and 360 min from the baseline scan.^34^ The MRI sequences utilized were reported in 29 studies (93%). Most of them were evenly distributed between: T2‐weighted spin‐echo (six studies),^2^, ^3^, ^4^, ^20^, ^32^, ^36^ balanced turbo field echo (eight studies),^21^, ^23^, ^25^, ^30^, ^34^, ^41^, ^44^ and balanced gradient echo (seven studies).^15^, ^22^, ^24^, ^37^, ^38^, ^42^, ^43^ The subject's position was reported in 27 studies (90%). In 20 of those (74%), the participants were positioned supine during the scan. In six (22%), a right decubitus position was chosen instead.^15^, ^24^, ^26^, ^35^, ^37^, ^39^

The caloric intake of the included meals ranged from 0 kcal (water)^1^, ^3^, ^23^, ^36^, ^44^ to 659 kcal (wholemeal bread, raspberry jam, and orange juice).^34^ In addition, all the included studies reported the consistency of the meals: 17 studies (56%) used liquid meals, 13 studies (44%) mixed meals, and no studies used exclusively solid meals. For a comprehensive overview of the included meals and their relevant nutritional proprieties (as reported by the respective authors), see Appendix 4.

In 15 studies (50%), a complete report of the meal composition could be found or calculated. As multiple meals could be applied in a single study, a total of 60 different types of meals, and their respective T50, were identified. Out of the 60 reported values of T50, the meal caloric intake was obtained in 59 (98%), fat percentage by weight in 52 (87%), and meal volume in 53 (89%).

**FIGURE 3:**
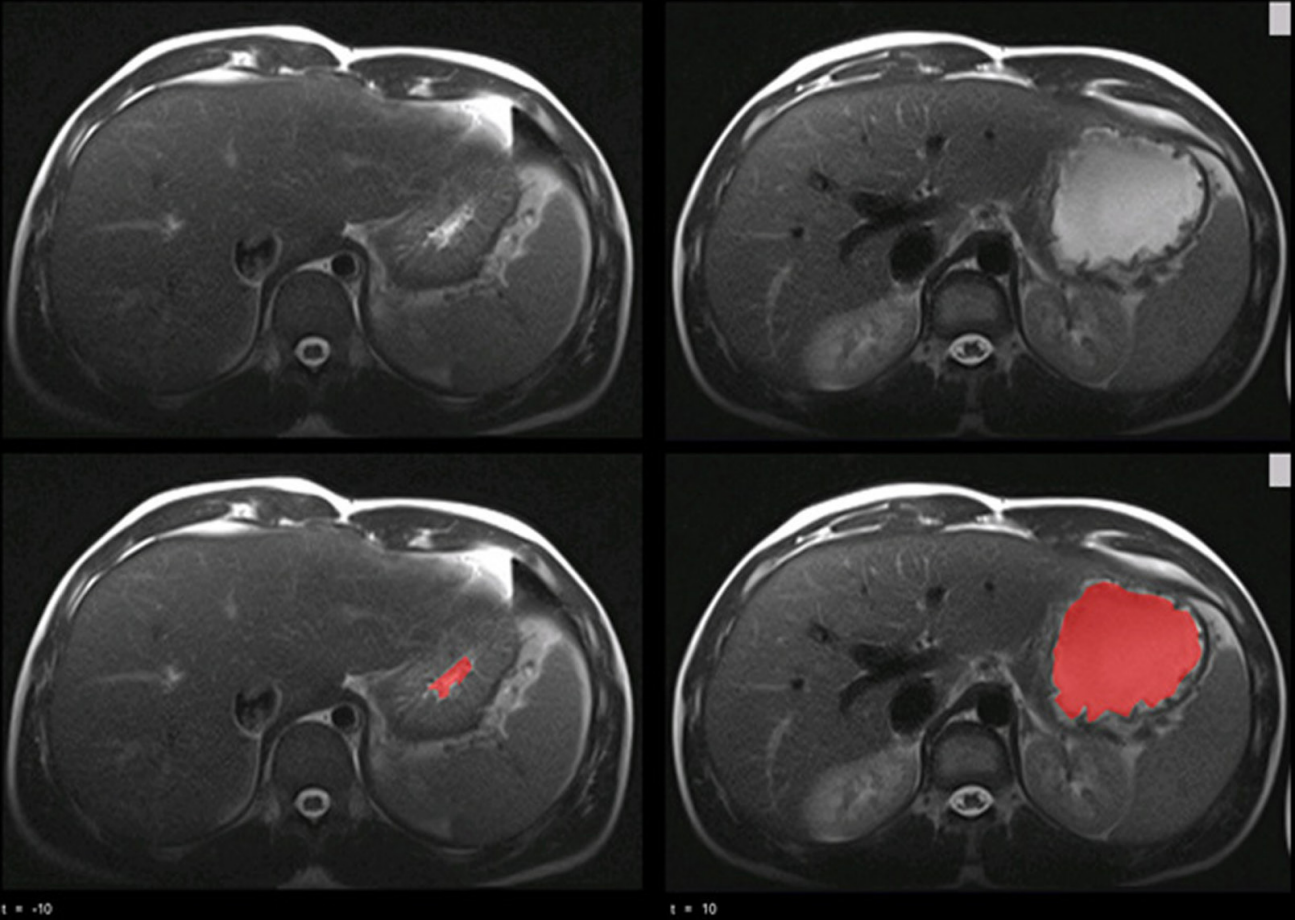
Example of volume segmentation in a T2‐weighted spin‐echo magnetic resonance image taken at the height of the liver, showing pre‐ingestion on the left and at 10 min post‐digestion on the right. In red, the segmented volume. © 2018 The Authors. Neurogastroenterology & Motility Published by John Wiley & Sons Ltd

The MRI volume assessments included in the calculation of the GE were obtained by manual segmentation in 19 studies (63%) and semi‐automatic segmentation in 11 (36%), see Figure 3 as an example of gastric segmentation.^47^ Of the latter, eight studies (73%) did not report how the boundary of the stomach was defined in the segmentation process.^1^, ^21^, ^25^, ^26^, ^27^, ^33^, ^38^, ^39^

### 3.6 GE assessment

The reported T50 ranged from 7 min of water (0 kcal, 240 ml) to 330 min of fat emulsion (530 kcal, 300 g). Of the included studies, one (4%) utilized the Elashoff model,^40^ three (12%) utilized a PowExp model,^1^, ^23^, ^44^ eight (32%) utilized a LinExp model,^15^, ^21^, ^25^, ^27^, ^31^, ^38^, ^39^, ^41^ five (20%) did not report the model utilized, while the rest (seven studies, 28%) utilized other models. For a comprehensive overview of the different T50 values and the other variables of the included studies, see Appendix 4.

### 3.7 Comparison with scintigraphy

Three articles were selected as our reference to identify the normal scintigraphic values.^5^, ^18^, ^48^ The reported normal T50 values were not always directly defined, as in one study, it was chosen to express the outcome as a percentage of retention at fixed timed points.^5^ For low‐fat mixed meals, the limits of a 50% retention (the equivalent of T50) were observed between 30 and 60 min (lower limit) and between 120 and 180 min (upper limit), with lower values suggesting rapid GE and greater values suggesting delayed emptying.^5^ An emptying half‐time of less than 25 min was reported for zero‐calories liquid‐only meals (water stimulus).^48^ In both articles, the amount of calories utilized was not directly stated but could be retrieved from their sources.^49^ A median emptying time of 88 min (range 49–146 min) was reported for liquid high‐fat meals (455 kcal).^18^ In our systematic review, all mixed meals T50 fell within the normal retention limits described using scintigraphy. All zero‐calorie liquid meals (six meals, 10%) fell within normal values. Four high‐fat liquid meals fell outside the standard value (13% of all high‐calorie liquid meals, 6% of the total).

## 4 DISCUSSION

As observed in this systematic review, the recent literature shows that GE can be assessed by MRI in healthy subjects with several protocols. Even though MRI protocols diverged in many aspects, they shared numerous elements and provided estimates of GE within the normal range, as defined by scintigraphy, when considering the inter‐method variability.^5^, ^48^

### 4.1 Overall study quality considerations

The quality of the included studies was quite heterogeneous, as many of them while having an overall sound quality, struggled to obtain satisfactory scores in the “Patient Selection” and “Index Test” domains of the quality assessment tool. In most studies, this was caused by incomplete or missing inclusion criteria or inclusion criteria dependent on the subjects’ self‐assessment, increasing the risk of possible bias. Furthermore, the composition of meals was often incompletely characterized or entirely missing. Another methodological weakness was the missing definition of gastric volume: An unclear anatomical boundary to the duodenum might result in inconsistent GE calculations, which could have been avoided by an *a priori* definition of the organs limits. While these minor variations might be negligible by themselves, they could be magnified by artifacts from respiratory and peristaltic motion, which further blur the organ's boundaries.^50^ These observations play an even more important role in automated segmentation techniques, which are already limited by the complexities of abdominal organs.^51^

### 4.2 Analysis of MRI protocols

#### 4.2.1 Inclusion and exclusion criteria of HVs

Most studies only included subjects ranging from 18 to 65 years. While this criterion can be justified by availability and feasibility issues, it complicates the results’ translation to the general population.^52^ To our knowledge, no previous article addressed this issue. Furthermore, almost all included studies relied on self‐reported health assessment to judge eligibility and inclusion. While this assumption can be credible, it might be a source of error. As previously published data would suggest, self‐reporting could lead to over‐ and under‐estimation, with varying grades of agreement depending on the specific symptom.^53^, ^54^ Suitability for MRI, no use of medication that could alter the gastrointestinal functions, and no evidence of gastrointestinal disorders were mandatory inclusion criteria in these studies. Exclusion criteria were often a history of bowel diseases or gastrointestinal surgical procedures, diseases that could alter the gastrointestinal functions, or hypersensitivity for any food products used in the experiments.

#### 4.2.2 Fasting protocols

Even modest levels of hyperglycemia (≥144 mg/dl or 8 mmol/L) can delay GE,^55^ and therefore, it appears to be the general consensus in scintigraphic studies that the blood sugar should be reasonably controlled on the day of examination.^5^ Furthermore, it is known that higher stomach volumes are related to higher GE rates. These observations suggest that a fasting period should be adopted on the scan day. While the length of this period can be discussed, it is not unreasonable to opt for longer fasting periods that should secure lower starting volumes and stable blood sugar levels. This is even more important for the translation to the clinical practice, where lower GE rates, for example, in gastroparesis, connective tissues diseases, and other metabolic or infiltrative disorders, could be magnified by additional meals influencing the assessment of residual stomach content.

The restriction for alcohol, caffeinated drinks, and strenuous exercise is undoubtedly worth mentioning and was implemented in approximately half of the included studies (15 studies of the 27 reporting fasting protocols, 55%). A smoking restriction was only found in two studies (7%), and thus, unrecognized activation of nicotinic acetylcholinergic receptors may have hampered GE time.^25^, ^27^

#### 4.2.3 MRI sequences and subject positioning

Different MRI sequences have shown the ability to visualize the gastric fluid content (and secretions, using gadolinium to enhance contrast). It appears that most sequences providing a good fluid/organ contrast, as most meals had a substantial water content, could be utilized for GE assessment, commonly by the use of a T2‐weighted sequence where fluids appear bright compared to surrounding tissue. Most of the studies included a baseline scan in their protocol and lasted longer than the reported T50, which guarantees an overall higher quality of the study outcome, mainly because the T50 is observed and not only estimated. The subject position in right decubitus was adopted in only a tiny percentage of the studies. This may cause a significant variability between different examinations, as previously published scintigraphy studies have suggested that different postures can lead to an error of up to 20% in the determination of emptying rate and to a reduction of emptying times up to 51%.^56^, ^57^ While those studies analyzed the differences between the lying position and the sitting‐standing position, which require a completely different technical setup during the image acquisition, it is an important detail to consider when deciding the patient positioning between scannings.

#### 4.2.4 Analysis and report of GE

In this systematic review, most studies used a manual segmentation method to extrapolate T50 from MRI images, with only a small percentage using semi‐automatic ones. These methods will not be discussed in detail here, but different segmentation methods may impact the assessment of T50. Briefly, manual segmentation methods require the operator to manually define the boundary of the organ of interest in each 2D image to obtain the total volume by their sum. On the contrary, most semi‐automatic methods require the user to manually place a region of interest in the target organ. Afterward, the boundaries of that organ are automatically generated through different algorithms, and the total volume is calculated by the sum of the obtained areas. Semi‐automatic methods are quicker, less operator‐dependent, and could be superior in terms of reliability by reducing intra‐ and inter‐rater variability.^58^ They are, however, reliant on the type of the defining algorithm.^59^

Most of the included studies report the GE in the form of T50. Some studies reported the GE as a series of volumes or emptied percentages at different time points. While a normal reference value of T50 has not yet been defined for MRI studies, we consider that T50 is clear, rapidly understandable, and easy to compare, and it should always be included in a report of GE, comprehending the algorithm utilized and the amount of data fed to it.

### 4.3 Comparison with scintigraphy

As expected, most of the included studies reported a T50 within normal range, as defined by the included reference articles. While all mixed meals and zero‐calories liquid meals fell within a normal range, a few high‐fat liquid meals fell outside of them (13%). This observation is probably due to the combination of high caloric content, high‐fat percentage, and relatively low meal volume utilized in their protocols.

### 4.4 Items that deserve further investigation

While many aspects of MRI protocols for GE assessment are getting progressively clearer, some aspects deserve further investigation:

#### 4.4.1 Preparation

It is still debated how long the fasting period should be or if different fasting periods should be adopted for solid or fluid meals, and it is yet to be investigated how pre‐meal gastric volume affects GE. Furthermore, the possible impact of the smoking restriction, which only the minority of studies adopted, should be investigated.

#### 4.4.2 Methods

The choice of meal is one of the essential factors in the emptying process, and it is worth assessing how all meal characteristics (including temperature, osmolality, and particle size) impact the GE.^60^ At present, a low‐fat “eggbeater” test meal is the most utilized in gamma scintigraphy investigations of GE. Unfortunately, because of the poor association between GE and GI symptoms and the inability of slow GE to predict the response to prokinetics,^61^, ^62^, ^63^ the performances of current GE studies are poor. Parker et al. hypothesized that the reason behind these poor performances is the small size of the meal, unable to trigger filling sensations, and have therefore validated the large “Nottingham Test Meal.”^27^

While this technique is not yet established and normal values from representative populations are still to be published, this work proposed a cheap and easy to prepare meal that would appear acceptable for both patients and clinicians, with only a few barriers for the implementation in clinical practice.^27^

As observed in this review, many MRI studies diverge in the investigation's length and the number of data points obtained. To help translate these techniques to the clinical setting, it is important to assess whether a “minimum data set” can be established. This would minimize the human and technical resources utilized in the image acquisition and outcome interpretation and, consequently, costs. Unfortunately, due to the lack of consensus in this field, a single (or multiple) standard summary outcome has not yet been defined, and it is therefore very challenging to assess the ideal duration of the examination. Furthermore, as new GE models are being implemented, the number of data points needed for the optimal accuracy of the algorithm utilized may vary. On the contrary, as newer segmentation methods are implemented, faster automatic methods will reduce the number of resources utilized in the post‐processing (as previously described) to observe or generate the chosen outcomes.

In most of the included studies, the HVs were scanned supine. It could be worth investigating the effect of right decubitus on GE, which some studies adopted.

#### 4.4.3 Report

In this systematic review, we chose to include only studies reporting T50 for ease of comparison and because it is currently the most adopted outcome measure. While this outcome parameter is clear and understandable, it may not alone describe a complex process like the GE. Fortunately, MRIs can provide a broad spectrum of different metrics that could participate in interpreting this process. Therefore, it would be preferable to include other summary outcomes, for example, volumes over time, emptying over time or residual volume at 2 h,^27^ to reduce the loss of information implied to the use of T50, providing a clearer view of the accommodative dynamic changes of this process, without overlooking the different phases of GE.^64^, ^65^

### 4.5 Conclusions

In accordance with our aims, we systematically compared how different MRI studies investigated the GE process. Even though we found several differences in many critical aspects of study designs, almost the entirety of the included studies reported outcomes within the normal range, as defined in the literature. These differences are not surprising, as all examinations consist of several components that can be combined with each other. While they do not appear to play a significant role in HVs, they reflect the necessity of obtaining a consensus in this field. Unfortunately, due to the number of studies included and to the selective inclusion of HVs, this review does not have enough evidence to suggest any form of best practice that could be translated into the clinical field except underlining the importance of a complete methodological report. In this kind of MRI examination, where every component can influence the dynamic of the whole process, those intending to start these studies must be familiar with the different variables described in this review to influence the decision‐making and guarantee their highest reproducibility and, therefore, their overall quality.

## DISCLOSURE

No competing interest declared.

## AUTHOR CONTRIBUTIONS

Davide Bertoli, Esben Bolvig Mark, and Jens Brøndum Frøkjær developed this review concept and protocol; Davide Bertoli and Emily Steinkohl were involved in data collection; Asbjørn Mohr Drewes, Davide Bertoli, Christina Brock, and Jens Brøndum Frøkjær directed the data interpretation; Davide Bertoli and Emily Steinkohl drafted the manuscript; Asbjørn Mohr Drewes, Christina Brock, Esben Bolvig Mark, and Jens Brøndum Frøkjær critically appraised the manuscript; Davide Bertoli wrote the manuscript.

## APPENDIX 1

Search strategy

Searching strategy for the included studies. For each database the full search strategy is reported. Every database was inspected the same day. Search date: 16.06.2020.

## APPENDIX 3

### QUALITY ASSESSMENT

Quality assessment tool for the included studies. Each study is graded with a Low/High/Unclear Risk of Bias and Low/High/Unclear concerns of Applicability.

## APPENDIX 4

**Table:**

Meal characteristics								
Study	Meal Calories (kcal)		Fat content (g)	Fat percentage (g/g total)	Volume (ml)	Weight (g)	T50 (min)	Meal type
Camps et al.^40^		500	24.1	5.5%‡	200	441	54.2‡	L
Camps et al.^40^		500	24.1	3.2%‡	500	741	29.6‡	L
Alyami et al.^41^		220	4.4	0.7%	640	§	45	M
Alyami et al.^41^		220	3.3	0.5%	640	§	47	M
Bawden et al.^3^		184‡	0.0	0%	250	§	44	L
Bawden et al.^3^		0	0.0	0%	250	§	17	L
Bharucha et al.^22^		296	§	§	296	§	99.3‡	L
Bluemel et al.^1^		430	10.0‡	§	§	†	110	M
Brianez et al.^24^		300	0.0	0%	600	§	48‡	L
Brianez et al.^24^		420	0.0	0%	600	§	45.6‡	L
Camps et al.^26^		100	3.0‡	0.6%‡	500	500‡	26.5	L
Camps et al.^26^		100	3.0‡	0.6%‡	500	500‡	41	L
Camps et al.^26^		500	15.0‡	3%‡	500	500‡	69.5	L
Camps et al.^26^		500	15.0‡	3%‡	500	500‡	81.9	L
Camps et al.^27^		500	17.7‡	2.9%‡	500	591	69.3‡	M
Carbone et al.^28^		§	§	§	400	§	101.8	M
Coletta et al.^29^		645	†	†	N/A	308	157	M
Coletta et al.^29^		659	†	†	N/A	308	151	M
Coletta et al.^29^		657	†	†	N/A	308	143	M
Fruehauf et al.^15^		§	§	§	200	§	104.9	L
Fruehauf et al.^15^		§	§	§	400	§	97.2	L
Fruehauf et al.^15^		§	§	§	600	§	85.6	L
Fruehauf et al.^15^		§	§	§	800	§	55.2	L
Gopirajah et al.^30^		224	0.9	0.2%‡	†	408	63.8	M
Gopirajah et al.^30^		373.2	9.2	2.3%‡	†	408	96	M
Grant et al.^42^		331	§	§	§	§	74	M
Grimm et al.^4^		0	0.0	0%	240	§	10.5	L
Grimm et al.^4^		0	0.0	0%	240	§	10.9	L
Grimm et al.^31^		103	0.0	0%	240	§	35	L
Grimm et al.^31^		102	0.0	0%	240	§	20	L
Grimm et al.^31^		102	0.0	0%	240	§	33	L
Grimm et al.^31^		0	0.0	0%	240	§	7	L
Hussein et al.^32^		526	59.4‡	19.8%‡	§	300	180	L
Hussein et al.^32^		526	42.6‡	14.2%‡	§	300	230	L
Hussein et al.^32^		526	42.6‡	14.2%‡	§	300	330	L
Khalaf et al.^33^		204	11.6	2.9%	§	400	44	L
Kuyumcu et al.^34^		450	§	35%	300	§	101	L
Kuyumcu et al.^34^		450	§	35%	300	§	107	L
Kuyumcu et al.^34^		450	§	29%	300	§	105	L
Mackie et al.^35^		237.6	7.8	2.9%	440	264	84	M
Mackie et al.^35^		237.6	7.8	2.9%	440	264	74	M
Marciani et al.^36^		532‡	3.9	0.9%	§	404	122	M
Marciani et al.^36^		435‡	14.3	3.8%	§	376	99	M
Marciani et al.^37^		241‡	12.0	2.7%‡	§	450	77	M
Marciani et al.^37^		241‡	12.0	2.7%‡	§	450	92	L
Marciani et al.^38^		541.8‡	5.8	1.7%‡	§	324	132	M
Marciani et al.^38^		537.5‡	7.4	1.1%‡	§	560	104	M
Menys et al.^39^		0	0.0	0%	300	300‡	20	L
Mudie et al.^43^		0	0,0	0%	240	240‡	13	L
Murray et al.^44^		110	0,2	0,1%‡	490	150	48	L
Murray et al.^44^		108	0,2	0.1%‡	490	150	39	L
Murray et al.^44^		110	0.2	0.1%‡	140	150	63	L
Parker et al.^20^		300	11.6	§	400	§	68	M
Parker et al.^21^		380	§	§	200	§	160	L
Parker et al.^21^		380	§	§	200	§	129	L
Parker et al.^22^		300	11.6	§	400	§	61	M
Shiraishi et al.^2^		50	0.0	0%	500	§	20‡	L
Min et al.^45^		200	6.0‡	§	400	§	64.1‡	L

Characteristics of the included studies: For each study, every different meal utilized is reported. In evidence: Caloric content (kcal), Fat content (g), Fat percentage (% of weight), Volume (ml), Weight (g), T50 (min), and thickness of the meal (L: liquid, M: mixed solid/liquid). §: no data reported, †: partial data reported (volume or weight dataset does not comprehend all the components), ‡: calculated from articles data.

**Table:**

T50 calculations				
Study	Type of Fit	Nr. of datapoints used		T50 (min)
Camps et al.^40^	Bayesan Fit. Linexp.		3	54.2/29.6
Bharucha et al.^24^	Bayesan Fit. Linexp.		5	99.3
Brianez et al.^25^	Bayesan Fit. Powexp, no overshoot.		2	48/45.6
Camps et al.^27^	Bayesan Fit. Linexp.		5	69.3
Shiraishi et al.^2^	Bayesan Fit. Linexp.		5	20
Min et al.^45^	Bayesan Fit. Linexp.		5	64.1

Fit models for the calculated T50: In evidence, the type of fitting curve utilized for the calculation, the number of datapoints used, and the obtained T50. The free online tool 'apps.menne‐biomed.de/gastempt/' was utilized for these calculations.
